# Novel Detection of Nasty Bugs, Prevention Is Better than Cure

**DOI:** 10.3390/ijms22010149

**Published:** 2020-12-25

**Authors:** Mia Strom, Tamsyn Crowley, Sarah Shigdar

**Affiliations:** 1 School of Medicine, Deakin University, Geelong 3216, Australia; mfstrom@deakin.edu.au (M.S.); Tamsyn.crowley@deakin.edu.au (T.C.); 2 Centre for Molecular and Medical Research, Deakin University, Geelong 3216, Australia

**Keywords:** aptamers, detection systems, hospital-acquired infections

## Abstract

Hospital-acquired infections (HAIs) are a growing concern around the world. They contribute to increasing mortality and morbidity rates and are an economic threat. All hospital patients have the potential to contract an HAI, but those with weakened or inferior immune systems are at highest risk. Most hospital patients will contract at least one HAI, but many will contract multiple ones. Bacteria are the most common cause of HAIs and contribute to 80–90% of all HAIs, with *Staphylococcus aureus*, *Clostridium difficile*, *Escherichia coli*, *Acinetobacter baumannii*, *Pseudomonas aeruginosa* and *Klebsiella pneumoniae* accounting for the majority. Each of these bacteria are highly resistant to antibiotics and can produce a protective film, known as a biofilm, to further prevent their eradication. It has been shown that by detecting and eradicating bacteria in the environment, infection rates can be reduced. The current methods for detecting bacteria are time consuming, non-specific, and prone to false negatives or false positives. Aptamer-based biosensors have demonstrated specific, time-efficient and simple detection, highlighting the likelihood that they could be used in a similar way to detect HAI-causing bacteria.

## 1. Introduction

Health and healthcare are important considerations in both the developing and developed world. The healthcare systems’ main purpose is to arrive unwell, gradually regain health and depart healthy. However, all over the world this is not always the case. Hospital-acquired infections (HAIs) are a major contributor to morbidity, mortality, and healthcare costs [1,2,3,4]. A HAI is an infection that occurs >48–72 h post-hospital admission [5,6] and are prevalent in both developing (incidence rate of up to 40%) [7,8] and developed countries (incidence of approximately 7%) [7]. Any patient admitted is at risk of developing a HAI, with 5–10% of all hospitalizations in Europe and North America resulting in a HAI [8]. However, those at most risk are those with weakened immune systems, such as burn patients, organ recipients, neonates, and intensive care unit (ICU) patients [5,7]. Patients in the ICU have a 5- to 10-times higher risk of developing a HAI due to many having a weakened immune system as a result of the use of mechanical devices such as catheters and ventilators [2,7,9,10]. It has been shown that infection and infection-related sepsis are the leading cause of death in non-cardiac ICUs with a mortality rate of 60% [2]. Due to the severity of some HAIs, they can lead to scarring and functional impairment as well as delays in recovery and death [2,5]. These delays in recovery lead to additional testing, surgeries and medicine, all cumulating in increased healthcare costs [2,3,5,11,12].

It has been shown that the socioeconomic status of the country or area is correlated with the incidence of HAIs. Lower socioeconomic status areas and developing countries have up to a 20-times higher incidence rate than higher income areas [7,10]. HAIs are caused by many pathogens, with viruses, such as human immunodeficiency virus (HIV) and influenza, making up approximately 5% of all HAIs. Fungi, such as *Candida albicans* and *Aspergillus* spp., also make up 5%, while bacteria by far are the most common cause of HAIs, accounting for approximately 80–90% of all HAIs [7,8]. The most common bacteria that cause HAIs include *Acinetobacter baumannii* [6,7,8,9,10,13,14,15], *Clostridium difficile* [8,13,16,17,18,19], *Escherichia coli* [5,6,7,8,9,13,20,21,22,23,24], *Klebsiella pneumoniae* [5,7,8,9,13,15,20,22,23,25], *Pseudomonas aeruginosa* [5,6,8,9,13,14,15,20,24,26,27,28,29,30] and *Staphylococcus aureus* [5,6,8,9,13,15,22,24,27,31,32,33]. Many bacteria that cause HAIs are commensal bacteria that become opportunistic pathogens upon moving to the infection site from their natural site [5,22,27]. This movement of commensal bacteria is typically triggered by the weakening of the host immune system via surgery, antibiotics, or open wounds [27]. S. *aureus* alone contributes to approximately 20,000 deaths in the US each year, which is a similar death toll to that of influenza, viral hepatitis, and HIV/AIDs [33]. A retrospective study conducted in 2019 showed that for 49.3% of all US hospitalizations; there were over 92,000 cases of *S. aureus* bacteriemia. Over 44,000 of these cases were attributed to methicillin-resistant *S. aureus* (MRSA). Furthermore, the *S. aureus* infections were closely associated with skin and soft tissue infections and pneumonia and contributed to a death rate of over 11,000 [34]. *C. difficile* contributes to 500,000 cases in the US, leading to 29,000 deaths. Due to this incidence rate, *C. difficile* is considered the leading cause of HAIs in the US [17,18]. This high rate of infection also leads to a high economic burden [3], with HAIs contributing to €1.5 billion per year in Europe and costing more than $4.8 billion in the US [15,17,18,35]. In addition, it has been shown that in 2016, in Europe, a prolonged length of hospital stay of up to 8 days accumulated an overall extra cost of €1,228,782 per year, which has increased with time [11]. 

## 2. Transmission, Resistance, and Persistence

The transmission of HAIs is one of the most important factors to be considered. It has been shown that the most common form of transmission is through person-to-person contact, via health care workers (HCW) or other patients, through the environment, contaminated water and contaminated surgical instruments [5,26,36,37,38,39,40]. *S. aureus* is a commensal bacteria that is typically found on the hands and nasal passages of many health care workers [41]. *P. aeruginosa* and *K. pneumoniae* are environmental bacteria that colonize the lungs and gastrointestinal tract, respectively. They are both known to colonize in moist [42], humid environments [38] such as sinks and drains, allowing for environmental transmission to patients through drinking water, showers, baths and even during surgeries [26,38,39]. *C. difficile* is also an environmental bacterium that typically colonizes the gastrointestinal tract after person-to-person transmission; a niche environment is formed due to an imbalance in the natural flora allowing for ease of colonization [16,19]. 

There are some ways to combat the spread of bacteria in the healthcare setting. It has been shown that with improved hand hygiene, such as using the CDC’s recommended “5 moments of hand hygiene” [5], that many infections caused by *S. aureus* and other HAI causing bacteria can be prevented [36]. Daily cleansing using no-rinse cloths saturated in 2% chlorohexidine has also been shown to decrease HAI incidence rate [1]. However, these methods only work to prevent some environmental spread.

The standard treatment for bacterial infection is the administration of antibiotics. Antibiotics were first discovered in the 1920s, and over the next 40 years, many new antibiotic discoveries were made. However, after the discovery of trimethoprim in 1968, the new discoveries began to dwindle. In the last 60 years, two new antibiotics were discovered, oxazolidinones in 2000 and lipopeptides in 2003 [43]. With the discovery and use of antibiotics came the emergence of antibiotic resistance in multiple bacteria. Antibiotic resistance is currently on the rise and many bacteria including: MRSA, *P. aeruginosa,* vancomycin resistant *enterococci* (VRE), and *K. pneumoniae* are known as multi-drug resistant [5,7,15,25,44,45,46]. Given the exponential rise of antibiotic resistance, it is becoming harder to treat bacterial infections, so we must turn our sights toward prevention. The development of better environmental detection systems could help to reduce the spread of bacterial infection by approximately 70% [3]. 

A major contributing factor to antibiotic resistance is that many of the highly resistant bacteria can form biofilms. A biofilm is formed when planktonic bacteria colonize a surface and some begin to breakdown forming a protective matrix composed of polysaccharides, proteins and lipids [21,47]. Antibiotics cannot penetrate the biofilm, and when inside a patient, complement and cell-mediated phagocytosis is also blocked [19,21,44,45,46,47]. Biofilms formed in the environment are also a concern, as bacteria in a biofilm are resistant to most detergents and disinfectants [37] and can survive in the biofilm for months [37]. Among the common HAI-causing bacteria, *S. aureus*, *P. aeruginosa*, *K. pneumoniae*, *E. coli* and *C. difficile* all form biofilms in response to harsh environments [19,48]. Biofilms are able to form on living and dead tissue, environmental surfaces and medical devices [19,46,47]. Due to their ability to form on internal medical devices, they are the leading cause of implant failure [19,44,45,46].

One of the major reasons why HAIs are so prevalent is the inherent difficulties in detection in the environment. If we were able to detect HAI causing bacteria more rapidly and efficiently in the environment, a minimum of 20% [49] and up to 70% of all HAIs could be prevented [3]. Some bacteria, such as *C. difficile*, can produce no symptoms, allowing for easier spread from person to person [17,19]. The isolation and detection of bacteria, like *C. difficile* that are known to have asymptomatic carriers could go a long way towards decreasing HAIs [17]. It has been shown that a rapid diagnostic, and by extension detection in the environment, decreases morbidity and mortality rates, thereby decreasing healthcare-associated costs [23]. The rapid detection, identification and isolation of pathogens are essential for public health protection [50,51]. 

Bacteria cause many HAIs including pneumonia, surgical site infections (SSI), urinary tract infection (UTI), bloodstream infections (BSI) and gastrointestinal (GI) system infections [5,6,7,8,13,14,16,17,18,19,21,25,29,33,42,45,52,53,54,55]. Table 1 shows common HAIs and their estimated overall incidence rates in hospitals. Among them, SSIs are more commonly caused by *S. aureus* [5,7], pneumonia is commonly caused by *P. aeruginosa* [13], and UTIs are commonly caused by *E. coli* [5,13,21]. 

Among these common HAIs, such as UTIs and pneumonia, there are more specific types of these infections. Catheter-associated UTIs and ventilator-associated pneumonia (VAP) make up the majority of UTI and pneumonia cases due to the presence of foreign materials in the body weakening the immune system [7,52]. This is also in part due to biofilms typically forming on inserted foreign bodies such as catheters and ventilators, with approximately 50% of all HAIs being due to indwelling mechanical devices [5,44]. It has been shown that 86% of all pneumonia is considered VAP [7,14], which is highly worrying due to the fact the *P. aeruginosa* and *K. pneumoniae* are both common causes of VAP. Both bacteria are highly resistant [35,56], with *K. pneumoniae* being colloquially termed a “collector” of resistance, typically gaining additional resistance via horizontal gene transfer from other resistant bacteria like VRE and *P. aeruginosa* [25,57]. More worrying still is the discovery that *K. pneumoniae* and *P. aeruginosa* can form multi-species biofilms, sharing resistances and protection [25]. It has been shown that, in these joint biofilms, neither bacteria outgrow the other, despite *K. pneumoniae* growing faster [25] than *P. aeruginosa* and that this multi-species biofilm is considered almost impossible to eradicate due to the high resistance of both species [19,25,44]. 

UTIs are typically caused by *E. coli* [8] and make up 150–200 million cases globally each year [52]. It has been shown that 40–50% of women and approximately 5% of men will contract a UTI in their lifetime, with many UTIs becoming recurrent [21,52]. It is estimated that due to the high incidence of UTIs, the US alone spends approximately $1.6–3.5 billion each year on UTIs [52].

This high number of HAIs is incredibly concerning due to the fact the most common cause of bacterial transmission is person-to-person contact [5]. It has been shown that the prolonged use of antibiotics can lead to the disruption of the natural flora within the patients’ microbiome, leaving a niche environment for the colonization of pathogenic bacteria, in particular *C. difficile* [52]. In many cases, these infections are quite complex, due to the presence of multiple HAI causing bacteria [5,7,8,9]. *C. difficile* is one of the leading causes of GI infections and the leading cause of health care-associated diarrhoea [16,17,18,19]. It is a highly resistant bacteria that can lead to increased mortality and increased transmission as it often presents as asymptomatic, causing it to be more difficult to detect and prevent spread [16,17,18,19]. The spread of these HAIs between patients via HCW or the environment is highly prevalent and can lead to increased mortality, morbidity, and costs. This ease of transmission for bacteria further highlights the need for an environmental detection system to attempt to reduce the prevalence of HAIs caused by bacteria [24]. 

## 3. Detection Systems

**Figure 1:**
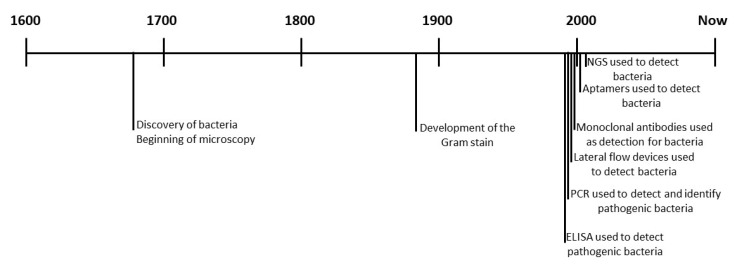
Timeline of development of bacterial detection systems. Figure 1 shows the timeline of the development of bacterial detection systems using different technologies, 1676—Discovery of bacteria and beginning of microscopy [68], 1883—development of the Gram stain [68], 1980—ELISA used to detect pathogenic bacteria [69], 1988—PCR used to detect bacteria [70], 1999—Lateral flow detection of bacteria [71], 1999—Monoclonal antibody detection of bacteria [72], 1999–2000s–Aptamer detection of bacteria [73], 2009—NGS detection of bacteria [74].

The current gold standard for detecting bacteria in healthcare is microscopy and cell culture [4,24,40,50,58,59,60,61,62,63,64,65,66,67]. This technique was first developed by Antony van Leeuwenhoek in the 1600s. Leeuwenhoek used the microscope to view the “tiny animals” that caused illness. As seen in Figure 1, for approximately 200 years, viewing under a microscope was the only detection system for microorganisms used and it was in 1883 that the next stage of bacterial detection was developed, the Gram stain [68]. The Gram stain is used to differentiate between Gram-negative and Gram-positive bacteria and allows for easier visualization of the morphological structure. This was an excellent breakthrough for the detection of bacteria and is still among the first points of call for bacterial detection today. Despite being the gold standard, microscopy does have some disadvantages. 

As seen in Table 2, culturing bacteria alone can take up to a week, it is a non-specific system, and can be prone to false negatives as many bacteria cannot be cultured or are in a dormant state, such as in a biofilm [4,40,59,60,61,63,64,65,66,75,76]. 

Microscopy remained the only detection system for bacteria until the 1980s when enzyme-linked immunosorbent assays (ELISA) were beginning to be used. An ELISA involves the use of a “capture” molecule and a “reporter” molecule; typically an antibody, to produce a readable signal which can be viewed on a plate reader [83]. As seen in Table 2, ELISAs are a relatively simple detection system [63], they have also been developed against a multitude of pathogens and can detect targets at a relatively low concentration [66,83]. However, ELISAs can be time consuming due to the need to pre-culture the bacteria and they rely on complex equipment and specifically trained personnel. ELISAs are also costly as they can use antibodies which are expensive to produce and are only single use [40,63,64]. 

Antibodies are small peptides that are a major component of animal immune systems. They are composed of a heavy and light chain and are developed by the immune system to specifically target foreign bodies. Antibodies bind to their targets via the induced fit model and are typically generated inside animal models when the animal has been exposed to the desired target. More recently, antibodies have also been shown to be selected through in vitro libraries, whereby the target is incubated with a randomized library of antibodies and any that bind are collected [89]. Antibodies typically have a Kd value in the nanomolar range, indicating high affinity binding and are currently being used for diagnostic purposes in ELISAs and other molecular diagnostic systems.

Following the use of ELISAs, as seen in Figure 1, came the development of polymerase chain reaction (PCR). PCR was revolutionary in detecting bacteria as the focus was on the specific bacterial DNA rather than the whole bacterium, allowing for the detection of smaller colonies [62,66]. PCR involves the replication and amplification of bacterial DNA using specific primers generated to bacteria specific sequences. The presence of the bacteria is then determined by running the PCR product on an agarose gel showing the amplification of the desired sequences. PCR is a simple process that can provide real time results. It is more specific as it can differentiate between bacterial species, which allows for a more tailored treatment for infected patients. Due to its reliance on bacterial DNA and specific sequences, there can be instances of false positives and false negatives due to incorrect sequence pairing and bacterial DNA mutations [62]. PCR has further been modified to include reverse transcriptase PCR and real-time PCR which, as seen in Table 2, increase the sensitivity of the assay but also increase the cost and time taken to complete [40,58,60,62,64,65]. PCR also does not distinguish between viable and non-viable cells, as only the presence of DNA is required; this lack of distinction can cause difficulties for detection purposes. Non-viable cells are irrelevant, while viable cells are the desired target, and without the differentiation, any detection test results could be irrelevant [65,66]. 

To continue with rapid detection sparked by the development of PCR came the development of next generation sequencing (NGS). NGS allows for the sequencing of the whole genome of an organism in a relatively short time. It is beginning to be used as a diagnostic tool in some hospitals and can identify the taxonomic species of infectious bacteria [59]. NGS is also able to provide a detailed analysis of complex systems, such as the human microbiome and seawater samples [59]. However, as seen in Table 2, NGS requires specialist equipment and a working knowledge of bioinformatics to interpret the data. NGS is also an expensive route due to the equipment, analysis involved and that the majority of NGS work is conducted off-site, although some hospitals are beginning to use it in-house [23]. 

Lateral flow assays (LFAs) were developed prior to NGS, however, there is much expansion in this area that could allow for them to be improved exponentially. LFA is a rapid, simple, cheap classic point-of-care (POC) detection method [63,90]. Typically, LFAs use a recognition element as a capture molecule [56], and use a signal-producing molecule as the detector to provide a readable signal that can be detected with the naked eye [40,90]. LFAs currently require a high concentration of bacteria to be effective and require some pre-culturing and pre-enrichment steps, however, they do have the potential to be improved upon allowing for the generation of a rapid, sensitive and specific POC test [60,90]. Antibodies are the typical recognition element used in LFAs, as such LFAs are “targeted” to specific bacteria and an appropriate target needs to be selected. 

Ideally for detection and diagnostics, a unique molecule, either expressed or secreted by the target organism/cell, is desired. These molecules could include surface proteins, lipo-polysaccharides (LPS), or toxins. Some bacteria, such as *C. difficile*, do produce toxins, allowing for them to be an ideal target for single bacteria detection [16,17]. However, as not all bacteria produce toxins, for multiplex detection systems, surface proteins and LPS are more ideal. LPS can be unique to some bacteria, however all Gram-negative bacteria have similar LPS, as do all Gram-positive bacteria, which would reduce the sensitivity and specificity of a detection system. Surface proteins are an ideal choice, as there are many that are specific to a particular species of bacteria, and there are even some surface proteins that are strain specific. Using bioinformatics pipelines allows for the identification of conserved and unique proteins [50]. Protein A (PA) is a conserved protein that is unique to *S. aureus* and it is used in the evasion of the host immune response. Given that antibody-based systems are generated using IgG antibodies which are the main antibodies used in the immune response, PA is not ideal for antibody-based detection systems; however, it could be used as a target for an aptamer-based system. Penicillin binding protein 2a (PBP2a) is also a conserved protein found only on MRSA strains, allowing for further specificity of detection [91]. Some bacteria, such as *C. difficile,* conserve non-host evasion surface proteins. For example, N-acetyl-muramoyl-L-alanine amidase is a conserved and unique surface protein to *C. difficile*, which is used to cleave the bacterial cell wall and membrane during replication. It is another protein that could be ideal as a target molecule for bacterial detection [92,93]. 

A study conducted in 2018 showed the use of polyclonal antibodies (pAbs). The pAbs were bound to a nitrocellulose membrane and were used to capture *Klebsiella* spp. Once the bacteria were captured, palladium nanoparticle labelled pAbs were added to produce a signal [94]. This test showed that the *Klebsiella* strains, including *K. pneumoniae* and *K. oxytoca* were successfully detected, as were *Roaoultella ornithinolytica*, *Serratia grimesii*, *Enterobacter aerogenes*, *Enterobacter cloacae* and *E. coli*. Given the false positive results of the detection of non-*Klebsiella* spp., this study also tested the urease activity of each culture, whereby only *Klebsiella* spp. were urease positive. This additional test of urease activity allowed for an extra element of specificity to detect only *Klebsiella* spp. and no others. The less specific nature of pAbs could be a reason for the false positive LFA tests. This LFA has a limit of detection of 10^4^–10^6^ cfu/mL, which is slightly less than PCR, NGS and ELISA [94].

A recent study, conducted in 2019, also showed the use of antibodies in an LFA to detect *Salmonella enteritidis*, however, this study had the novel addition of using an antibiotic as an additional recognition molecule [90]. Due to the mechanism of action of ampicillin (AMP), a beta-lactam antibiotic which binds to the cell wall of bacteria, it can be used as a “capture” molecule, and in this study, was also conjugated with a magnetic nanoparticle to isolate the “caught” bacteria. The antibiotic-bacteria complex is then immobilized by binding to the monoclonal antibodies bound to the test line of the LFA. It was shown that the antibody and the AMP each bind to different recognition sites on the surface of *S. enteritidis* allowing for ease of dual recognition [90]. Due to the use of antibiotics which bind non-specifically to many bacteria, this could be a good POC detection system for environmental bacteria. The system also relies heavily on a specific “reporter” molecule, in this case the antibody. This system has a 30-min reaction time and a limit of detection of 10^2^ to 10^7^ cfu/mL, highlighting the rapidity and sensitivity of LFAs [90]. 

LFAs can be highly modifiable to detect multiple targets in a single test, whereby each capture molecule is at a different site along the strip or using a different colored reporter molecule per infectious disease, creating a multiplex detection system [63,95]. This technology could be utilized to determine multiple HAI causing bacteria within the hospital environment [63]. A study conducted in 2017 by Scharinger et al. showed the detection and serotyping of *Cronobacter sakazakii* a bacterium responsible for sepsis, meningitis and necrotising enterocolitis in infants [96]. This detection system utilized highly serotype specific monoclonal antibodies in a multiplex lateral flow format [40]. The samples were first incubated with the specific antibodies, whereby the 1C4 antibodies, serotype O1 specific, was digoxigenin (DIG) labelled and dinitrophenyl (DNP) labelled to form a sandwich complex with the antibodies binding to an LPS structure on the bacterial surface. The 2F8 antibodies, serotype O2 specific, were DIG- and biotin-labelled, creating a different sandwich complex. The LFA strip was lined with anti-DNP antibodies, test line 1, and streptavidin, test line 2. The serotype O1 sandwich complex would bind to test line 1, while the O2 complex would bind to test line 2. Finally, gold labelled anti-DIG antibodies would bind to the DIG-labelled antibodies on both test line 1 and test line 2, showing a visible red band, with any additional anti-DIG antibodies binding to the control line, a goat anti-mouse antibody. The presence of a red line at test line 1 or 2 with the red line at the control would indicate the presence of *C. sakazakii* serotype O1 or O2 respectively. The presence of red lines at all test lines indicated the presence of both serotypes, and a red line at only the control indicates a negative result. The specificity was tested using multiple strains of the O1 and O2 serotypes, as well as using O3, O4 and O7 serotypes, multiple other *Cronobacter* spp. strains and three additional *Enterobacteriaceae* family members. Only the O1 and O2 strains showed colored test lines 1 and 2 respectively, all other strains only showed a colored control line. The entire process took approximately 15 min and has a limit of detection of approximately 10^8^ to 10^9^ cfu/mL [96]. Due to the quick processing time, it is an ideal example of how an LFA could be used as a rapid POC detection system.

This multiple style assay can also be used for multiple species of bacteria, as shown in a study conducted by Zhao et al. in 2016, showing that ten common foodborne bacteria were detected specifically in a single LFA. The individual strips for the ten bacteria were combined in a disc using an up-converting phosphor (UCP) as the reporter molecule [63], whereby the sample would be added to the center of the disc and would travel outwards along the strips to generate the results via antibody binding. The limit of detection of the strips was determined to be 10^4^ to 10^5^ cfu/mL depending on the target. It was determined that the multiplex and single assays for 6 of the bacteria did not differ in sensitivity. However, the single assays for *E. coli* O157:H17, *Salmonella chloeraesuis*, *Vibrio cholera* O1, and *Vibrio cholera* O139 were more sensitive than the multiplex assay for those species [63]. The differing running buffers used were attributed to the change in sensitivity. This was due to differing running buffers used in the single assays, while only one running buffer could be used in the multiplex assay.

One of the major issues of using LFAs as a detection system is the lack of efficient quantitative analysis. The simple presence or absence test lacks the information of how much of a target is present. A study conducted in 2018 aimed to resolve this issue. Wang et al. utilized a surface-enhanced Raman scattering (SERS)-based LFA strip, which allowed for both qualitative and quantitative analysis [97]. The assay was used to determine the presence of three high rank bioterrorism agents, *Yersinia pestis*, *Francisella tularensis* and *Bacillus anthracis*. Each of these bacteria have contributed to high mortality and morbidity rates and are “Category A” bio-threat agents, which is the highest rank for potential bioterrorism agents. The SERS-based LFA utilizes the typical LFA setup, in that there is a test line, with an antibody specific to the target and control line, non-specific to the target. However, during the assay, the Raman intensity of a characteristic Raman peak is monitored allowing for qualitative analysis. Instead of AuNPs, Raman antibody-labelled AuNPs of an increased size were used [97]. The assay took 15 min and the limit of detection for this assay, to be viewed by the naked eye was determined to be 10^5^ cfu/mL for all three bacteria. For the SERS-based assay, the limit of detection was 43.4, 45.8 and 357 cfu/mL for *Y. pestis*, *F. tularensis* and *B. anthracis,* respectively. These low values are estimated to be approximately three to four orders of magnitude more sensitive than colorimetric LFAs. Each strip was tested with each bacterium, showing *Y. pestis* only binding to the *Y. pestis* specific strip, with both *F. tularensis* and *B. anthracis* only binding to their respectively strips also. These results showed specificity between each LFA test [97]. 

LFAs could be a good detection system for bacteria in the environment given their rapid runtime and simplistic setup. LFAs are not without fault however, as seen in Table 2: LFAs can be prone to false binding causing false positive results and, in some cases, require specific equipment to view the results. An alternative or potential addition to LFAs are biosensors. Biosensors were also developed prior to NGS and have had rapid expansion in the field like LFAs. Biosensors are devices that incorporate a recognition element [62,64,95,98,99,100]. They also incorporate a transducer which generates a measurable signal, which can be optical, electrochemical, or mechanical [24,60,62,64,95,99,101]. Biosensors can be utilized in conjunction with LFAs, and in some cases, an LFA can be classified as a biosensor. 

There are, however, some disadvantages to using antibodies. They can be expensive to generate due to the use of animals, they can be prone to degradation at varying temperatures and pH levels and have been known to have variability between batches [64,65,81]. Traditional antibody generation can be difficult for some bacteria, as antibodies have been known to have difficulty detecting certain bacterial species. There are many pathogenic bacteria that have specific surface proteins to evade the host immune system. This can be done by non-specifically binding to the Fc regions, making IgG antibodies unusable for those bacteria. Leading to false positives due to the presence of the single proteins without detecting the bacteria itself. This issue can be counteracted by utilizing IgY antibodies found in chickens and reptiles [24] or through the use of antibody libraries used in in vitro selection. It has also been shown that antibodies will bind preferentially and sometimes non-specifically to Gram positive bacteria, which are highly abundant in the environment and can lead to false positive results [58]. An alternative to antibodies is aptamers, colloquially termed “the chemical antibody” [50]. Aptamers are single stranded oligonucleotides that fold into a three-dimensional conformation and bind via the induced fit model like antibodies. They are generated synthetically via a process known as systematic evolution of ligands via exponential enrichment (SELEX) [4,30,81,84,102,103,104]. A randomized pool of oligos is incubated with the desired target, and those that bind are amplified via PCR and the process is repeated 7–12 times to achieve an enriched pool of specific and sensitive binders [82]. There is typically also a negative selection step to increase specificity, whereby a similar target molecule is incubated with the pool and those that do not bind are collected and amplified to remove any weak or non-specific binders [56]. Aptamers have the advantage over antibodies in that they are more stable at varying temperature and pH levels, can be generated to a wide range of targets, including those that do not generate an immune response, such as toxins [50,82,88,100,102], as well as having comparable levels of specificity and sensitivity to antibodies [4,30,50,81,88,102,103].

**Figure 2:**
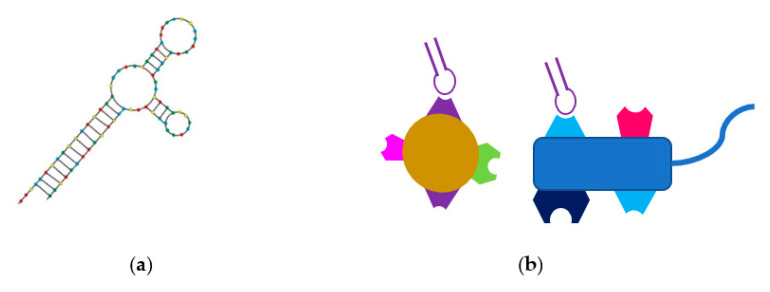
Schematic representation of a single aptamer and a bi-reactive aptamer. (**a**) A representation of a single aptamer (**b**) A schematic of a single aptamer able to bind to two different bacterial species [87].

In 2018, Zou et al. generated an aptamer to bind specifically to *Enterohemorrhagic E. coli* strain O157:H7. This aptamer Apt-5 was selected using whole-cell SELEX, whereby the randomised library was incubated with the whole bacterial cell rather than a single protein, peptide or LPS. Apt-5 has a Kd value in the nanomolar range and showed a low binding rate to bacteria other than *E. coli* [105]. Additionally, as seen in Figure 2, aptamers have also been shown to be able to bind to multiple targets by modifying the SELEX process, whereby with each incubation step, the target is toggled between each of the multiple targets, allowing for the generation of an enriched pool of specific aptamers that bind to multiple targets [87,106]. This process is known as Toggle SELEX. 

A study conducted in 2017 by Song et al. showed the generation of a single aptamer that bound specifically to six different bacterial species [87]. Song et al. utilized the toggle SELEX method by sequential “toggling” between each target in each incubation step. In this experiment, *E. coli*, *K. pneumoniae*, *E. aerogenes*, *Bacillus subtilis*, *Citrobacter freundii* and *Staphylococcus epidermis* were each incubated with the aptamer library. First *E. coli* then *E. aerogenes* and so on until *S. epidermis*. This cycle was repeated three times to ensure accurate binding to all six bacteria. Toggle SELEX could be an ideal method for the generation of a highly specific detection system for environmental bacteria [87]. Currently, there are many aptamers already characterized that could be utilized in an LFA or a biosensor in many industries, including hospitals. Table 3 shows some of the current characterized aptamers to different bacteria. The authors acknowledge that not all generated aptamers have been listed but some seen in Table 3 could be utilized for better detection systems in multiple industries. 

Most of the aptamers in Table 3 have been characterized using flow cytometry to determine the Kd value of the aptamers. Some, such as A11, ML12, ML6, ML7, JN17, JN21, JN08 and JN27, have been further characterized through additional experiments. A11 was utilized in an enzyme linked apta-sorbent assay (ELASA) to determine if it influenced cytokine production in peripheral blood mononuclear cells [120]. The experiment showed that A11 did reduce cytokine production, however this was not due to a cytotoxic effect on the cells, but due to binding to the staphylococcal enterotoxin preventing its action. Wang et al. showed that the aptamer could be modified by chelating PEG to the 5′ end, which did not interfere with binding. This PEGylated A11 contributed to a 90% recovery from toxic shock syndrome in a mouse model [120], showing the use of aptamers, not only as a diagnostic, but also as a vehicle for treatment.

The M12 aptamer was also further characterized using an ELASA method, whereby the aptamer was biotinylated and visualized using the yellow coloring of TMB. It was shown in this characterization that the M12 aptamer showed varying strengths of the yellow color, corresponding to the aptamer concentration, while the ML6 and ML7 aptamers had no color development [107]. It was hypothesized that ML12 mechanism of action could be as a competitive binder, thereby blocking the *B. anthracis* lethal factor from cleaving MEK1. This was tested via running the samples on a native gel with a specific line showing the presence of lethal factor and a line showing MEK1 [107]. With increased addition of the ML12 aptamer, there was an increase in strength of the MEK1 band, indicating that the aptamer was preventing the cleavage of MEK1 by acting as a competitive inhibitor. A cell viability assay was also conducted, showing that the cell viability was not significantly affected by the presence of the aptamer [107]. 

Soundy et al. developed four aptamers that could be used as diagnostic tools. They took a different additional characterization approach by determining if the JN17, JN21, JN08 and JN27 aptamers had bacteriostatic or bactericidal effects to test if the aptamers could be used as a diagnostic and therapeutic or, theragnostic [117]. The metabolic activity of the bacteria was determined by adding 5-cyano-2,3-ditolyl tetrazolium (CTC) to the LB broth, as in the presence of metabolically active cells CTC is oxidized to an insoluble precipitate [117]. To determine if the aptamers were producing a bactericidal effect the optical density of the LB broth was determined via plate reader. Soundy et al. concluded that none of the aptamers were bactericidal or bacteriostatic [117]. 

**Figure 3:**
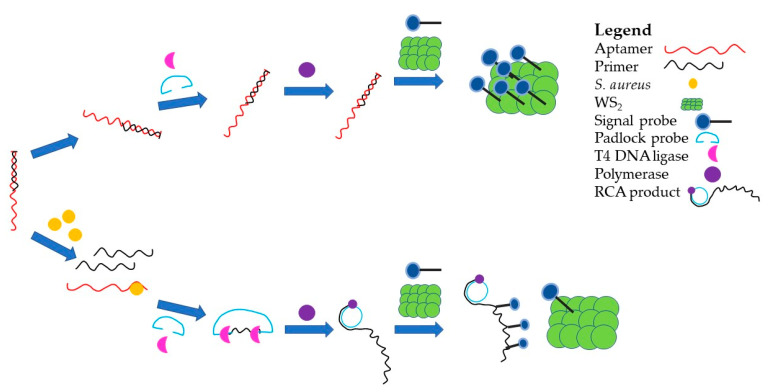
A schematic representation of an aptamer-based detection system developed by Hao et al. [67]. A representation of an aptamer-based detection system utilizing RCA, whereby an aptamer is bound to an RCA primer. When in the presence of *S. aureus*, the aptamer binds preferentially to the bacteria releasing the primer to begin RCA. The formation of the RCA product acts as a target for an added signal probe, creating a readable signal. In the absence of *S. aureus*, the primer is not released and the RCA product is not created, which leads to the signal from the probe being quenched by a WS_2_ nanosheet [67].

There have been examples of bacterial detection systems incorporating antibodies or aptamers as the recognition element and gold nanoparticles (AuNP) as the transducer to produce an optical signal. The use of gold nanoparticles (AuNP) as the transducer in biosensors is a common theme due to their ease of generation and ease of use [58,99]. Figure 3 shows a schematic of one such study, which showed the detection of bacteria using a Co^2+^ enhanced N-aminobutyl-N-ethylisolumiol functional flower-like AuNP (Co^2+^/ABEI-AuNF) as a donor, a WS_2_ nanosheet as the acceptor and rolling circle amplification (RCA). This detection system involved an aptamer/primer complex where, when exposed to *S. aureus*, the aptamer preferentially binds to the bacteria releasing the primer sequence and allowing the beginning of RCA. Once the RCA occurs, T4 DNA ligase and DNA polymerase allows the binding of the RCA product to the Co^2+^/ABEI-AuNF instead of the WS_2_ nanosheet. In the absence of *S. aureus*, the primer sequence is not released, therefore the RCA product is not produced, allowing the Co^2+^/ABEI-AuNF to bind to the nanosheet, thereby quenching the nanoparticle signal [67]. 

Another example where aptamers have been used to detect bacteria, specifically *S. aureus,* utilized two aptamers, one binding to PA, a unique and conserved protein found on all strains of *S. aureus*, and one aptamer binding to PBP2a, a protein found only on MRSA [24,33,53,91]. The PA aptamer was conjugated with streptavidin magnetic beads to capture any bacteria expressing PA [56], which includes both MRSA and standard *S. aureus*. The PBP2a specific aptamer is conjugated with a blocker sequence and when exposed to MRSA, it detaches from the blocker and preferentially binds to the PBP2a protein. Once the blocker is released it binds to the padlock completing the circular DNA complex allowing for activation of RCA using Cas12a. This activation then allows a CRISPR enzyme to cleave the product which contains Cy3, unquenching the fluorescent molecule [91]. Aptamers can also be used in label free detection as certain nucleic acid dyes can be used to determine if the aptamers have bound to a target. Methylene blue (MB) is an aromatic cationic dye that has optical and electrochemical properties. MB typically binds to DNA via intercalation in between two guanine bases. By adding G-C bases pairs in the stem of the bacteria specific aptamer, MB could be added as an electrochemical label free detection, by viewing the change in peak density using a UV spectrophotometer [100]. In 2018, a study conducted by Brosel-Oliu et al. showed the generation of an aptasensor to selectively detect the presence of *E. coli* O157:H7. This aptasensor was developed using electrochemical impedance spectroscopy, whereby a three-dimensional interdigitated electrode array was coated in mercaptosilane. The *E. coli*-specific aptamer was tagged with a disulphide molecule allowing for a thiol/disulphide exchange reaction to bind the aptamer to the array [64]. This binding of *E. coli* to the aptamer produced an impedimetric change readable by the impedance sensor. This entire assay took approximately 30 min and had a limit of detection of 10^2^ cfu/mL [64]. Given the short time taken and low limit of detection, this assay could help to pave the way for an easy and efficient POC bacterial detection system. 

As previously mentioned, toggle SELEX could be an ideal method to generate a multiplex detection system for HAI causing bacteria, as evidenced by a recent study showing the development of an aptasensor that could differentiate between influenza strains [56]. The aptamers are biotinylated and have a high specificity to a single strain of the H3N2 virus. To determine binding specificity, the aptamers were compared to commercially available antibodies, labelled with AuNPs. The process of adding samples and completing imaging took approximately 15 min, indicating that this could be an ideal POC detection system. The antibodies showed binding to the desired strain as well as two other strains of H3N2, while the aptamer only showed binding to the desired strain. These results show an increased specificity in the aptamer over the antibody and demonstrates that potentially current biosensors using antibodies could be improved with the addition of aptamers [56]. The aptamer was able to differentiate between the different strains due to the inclusion of a negative selection round during the SELEX process [102,130]. The negative selection round allows for the reduction of cross-reactivity between the final aptamers and undesired targets. By employing this round when selecting for HAI causing bacteria aptamers could be selected to bind specifically to infection causing bacteria only while ignoring bacteria that are important for the environment and within the human microbiome. The negative selection round could be further improved upon by utilizing a well-known technique of heterologous protein expression [131]. By determining unique and conserved proteins for the bacteria and transfecting those proteins into a benign undesired lab strain bacterium, the selection process could be greatly enhanced. Incubating the multiple transfected bacterial strains with the aptamer library and then incubating with the wild type strain could allow for the removal of non-specific binders and ensuring the aptamers bind to the desired bacterial proteins in a biological relevant state. This could also be included in a toggle SELEX method, allowing for multiple bacterial proteins to be used one after the other as per toggle SELEX [87], while maintaining a strong negative selection round, allowing for the reduction of non-specific binding.

The previously mentioned studies all show rapid POC detection with high specificity. Each detection takes between 15–30 min and has a limit of detection ranging up to 10^9^ cfu/mL. By far the most sensitive detection system was the Raman SERS-based LFA having a limit of detection of as low as 43 cfu/mL for *Y. pestis* when using the SERS technology. The naked eye detection was also quite low at 10^5^ cfu/mL. This study has shown some of the lowest limit of detection values for bacterial detection LFAs. The incorporation of the SERS-based assay has shown to increase sensitivity however also increase cost and the need for specialist equipment, reducing its ability to be used as a POC. The difficulty is finding an appropriate balance between ease of use and sensitivity [50,51,60]. Both the AMP-antibody *S. enteritidis* and aptamer-based impedance detection systems took 30 min, with a low limit of detection of 10^2^ cfu/mL [64,90]. Both assays were relatively cheap and could be used as ideal POC detection systems. The influenza aptasensor showed that aptamers can have a higher specificity than antibodies, indicating that with the addition of aptamers in currently used antibody-based LFAs or biosensors, the specificity could be significantly increased [56]. Compared to the current detection methods all assays mentioned are more sensitive than PCR and ELISA, which have an approximate limit of detection of 10^3^ cfu/mL [80]. However, both assays take much longer time than the previously mentioned biosensors and LFAs; PCR taking up to 2 h and ELISAs taking up to 3 h. NGS also has a high sensitivity however, in some cases has been shown to have a lesser sensitivity than PCR or basic culture [132] and can take 24–48 h [133]. Given the rapidity and ease of use of biosensors and LFAs, they are an ideal choice for bacterial detection in the environment. The use of these rapid and cheap POC tests could be used to determine environmental sources of infection and the effectiveness of current hospital cleaning practices. Aptamers and antibodies are both excellent choices for recognition molecules [56,63,128], however, given that aptamers are more stable at varying temperatures and pH levels, and have been shown to be more sensitive than many antibodies, they would be a better choice moving forward. Not only has the influenza assay shown that aptamers can be more sensitive [56], but they are more easily modifiable during selection and can easily be generated to specifically target multiple bacteria with a single aptamer [87], allowing for cheaper and easier POC systems. 

## 4. Conclusions

HAIs are responsible for high mortality and morbidity rates as well as contributing to a high economic burden in developed and developing countries [7]. The introduction of a portable and rapid POC detection system could greatly reduce these rates, potentially preventing up to 10–70% of all HAIs [3]. It has been shown that with the addition of target “capture” and “reporter” molecules such as aptamers, the sensitivity and specificity of already established detection systems is increased. There is an immediate need for highly sensitive and specific POC detection systems on the surfaces of hospitals as well as on the hands and clothes of HCW. The spread of highly infectious HAI-causing bacteria is directly linked to the environment and person-to-person contact with HCW. These detection technologies could also be used in areas other than bacterial detection. The recent COVID-19 pandemic has shown the need for rapid, sensitive, and specific detection systems with real-world applications [60,134]. By developing a POC test against COVID-19 many patients who are waiting up to a week for results could know in a matter of hours, reducing chances of spread and potentially increasing chances of survival [134]. A POC test using aptamers could be in a LFA format due to them being cheap, quick and the results are easy to interpret [40,56]. A biosensor detection system could also be used, allowing for an optical fluorescence or an electrochemical readout [66]. As mentioned previously, an aptasensor was developed to distinguish between different species of the H3N2 influenza virus [56] and this technology could easily be utilized in both HAI-causing bacterial detection and the COVID-19 pandemic. Regardless of which system is used the path ahead is clear. A rapid, portable, POC detection test is needed and could be used not only in hospitals, but also, the food industry, agriculture, environmental monitoring and homeland security and defense [60,135]. 

## Figures and Tables

**Table 1:** Common hospital-acquired infections and the most common bacteria associated with them [5].

Infection	Overall Percentage (%)	Most Common Organisms (%)
Surgical Site Infections	(19.6%)	*Staphylococcus aureus* (17.9%)
Pneumonia	(19.4%)	*Pseudomonas aeruginosa* (17.4%)
Urinary tract infections	(19%)	*Escherichia coli* (36.2%)
Bloodstream infections	(10.6%)	Coagulase-negative *Staphylococci* (18.5%)
Gastrointestinal system infections	(7.6%)	*Clostridium difficile* (48%)
Other Lower Respiratory Tract Infections	(4.1%)	*Staphylococcus aureus* (12.6%)
Other infections	(19.7%)	Unspecified

Table 1 shows that surgical site infections (SSIs), pneumonia and urinary tract infections (UTIs) are the most common hospital-acquired infections (HAIs). *S. aureus* is the most common cause of SSIs and other lower respiratory tract infections. *P. aeruginosa* is the most common cause of pneumonia, *E. coli* is the most common cause of UTIs, coagulase-negative *Staphylococci* are the most common causes of BSIs and *C. difficile* is the most common cause of gastrointestinal infections.

**Table 2:** Detection and diagnostic systems currently used and their advantages and disadvantages [65].

Detection and Diagnostic System	Aptamer or Antibody Applicable	Advantages	Disadvantages	Location and Limit of Detection
Culturing and microscopy	Neither applicable	Detects presence of bacteria Easy technique Does not require specialist equipment Relatively cheap	Some bacteria are un-culturable Prone to false negatives Lack specificity—only detects presence or absence not species, which is not desired for a diagnostic Time-consuming [24,39,58,60,61,62,76,77,78]	Pathology laboratory Limit of detection: N/A, time is the factor rather than concentration, the bacteria will grow but will take longer with a lower cfu/mL
ELISA	Both applicable	Specific Little chemical preparation required Cheaper	Expensive equipment Requires specialist equipment Time-consuming Requires culturing [58,60,61,62,77,79]	Pathology laboratory Limit of detection: 10^4^–10^6^ cfu/mL
PCR	Neither applicable	Requires small amount of bacteria Specific—can identify species Easy technique Does not require specialist equipment	Requires specific probes Point mutations in bacterial genes can lead to false negatives and false positives Time-consuming Expensive [62,80]	Onsite or pathology laboratory Limit of detection: 10^3^ cfu/mL
Real time PCR	Neither applicable	Time-efficient Requires small amount of bacteria Specific—can identify species	Requires specific probes Point mutations in bacterial genes can lead to false negatives and false positives [62,80]	Pathology laboratory Limit of detection: 10^3^ cfu/mL
Next generation sequencing	Neither applicable	Time-efficient Requires small amount of bacteria Specific	Requires specialist equipment Requires bioinformatics knowledge [59]	Sequencing company Limit of detection: 10–100 cfu/mL
Biosensors (Antibodies)	Antibody	Highly specific (nanomolar) Time-efficient	Batch-batch variation Expensive Prone to steric hindrance Degrades in heat and pH changes Can cause immune response [24,60,61]	Onsite or pathology laboratory Limit of detection: 10^3^ cfu/mL
Biosensors (Aptamers)	Aptamer	Highly specific (nanomolar to femtomolar) Time-efficient High signal density Low steric hindrance Easily modifiable Cheaper Does not use animals Does not degrade in high heat or changing pH Reusable No immune response	Nuclease degradation Can be too small [24,81,82,83,84,85,86,87,88]	Onsite Limit of detection: 10^2^ cfu/mL
Lateral flow devices	Both applicable	Time-efficient Cheap Simple	Can be prone to false binding Can be complex to use Can require complex equipment [63]	Onsite or pathology laboratory Limit of detection: 43 cfu/mL to 10^9^ cfu/mL

Table 2 shows various diagnostic and detection methods used currently. It also shows each methods’ advantages and disadvantages as compared to the other methods.

**Table 3:** Aptamers currently generated against bacteria.

Aptamers	Type of Aptamer	Organism	Target
ML6, ML7 and ML12	DNA	*Bacillus anthracis* [107]	Lethal factor
PA1	DNA	*Bacillus anthracis* [108]	Protective antigen
ONS-23	DNA	*Campylobacter jejuni* (strain A9a) [109]	Whole bacteria
P12-31	DNA	*Escherichia coli* (ATCC 25922) [110]	Whole bacteria
EA1 and EA7	DNA	*Escherichia coli* (strain 11775) [4]	Whole bacteria
8.10A, 8.14B, 8.18B and 8.28A	DNA	*Escherichia coli* DH5α [111]	Whole bacteria
AM1, AM2, AM3, AM4, AM5 and AM6	DNA	*Escherichia coli* O157:H7 [112]	Whole bacteria
Apt-5	DNA	*Escherichia coli* O157:H7 [105]	Whole bacteria
Hp4	DNA	*Helicobacter pylori* [113]	Whole bacteria
hemag1, mag1 and hemag3	DNA	*Lactobacillus acidophilus* (strain 4355, 4356, 4357) [103]	Whole bacteria
Antibac1 and Antibac2	DNA	Multiple species [114]	Peptidoglycan
Mtb36	DNA	*Mycobacterium tuberculosis* (strain H37Ra) [115]	Whole cell
NK2	DNA	*Mycobacterium tuberculosis* (strain H37Rv) [116]	Membrane proteins
JN17, JN21, JN08 and JN27	DNA	*Pseudomonas aeruginosa* [117]	Whole bacteria
33	DNA	*Salmonella enterica* serovar Typhimurium [116]	Outer membrane proteins (OMPs)
S-PS8.4	RNA	*Salmonella enterica* serovar Typhimurium [116]	Type IVB pili
ST2, ST3, ST7 and ST9	DNA	*Salmonella typhimurium* (strain ATCC 50761) [118]	Whole bacteria
C5, C7, C10, C13 and C16	DNA	*Staphylococcal* Enterotoxin A [119]	*Staphylococcal* Enterotoxin A
A11	DNA	*Staphylococcal* Enterotoxin B [120]	*Staphylococcal* Enterotoxin B
SA20, SA23, SA32, SA34 and SA43	DNA	*Staphylococcus aureus* (strain MRSA) [116]	Whole bacteria
PA#2/8, PA#2/8[S1-58], PA#2/8[S1-50], PA#2/8[S1-43] and PA#2/8[S28-50]	DNA	*Staphylococcus aureus* [121]	Protein A
Pa-C10 and PA-C8	DNA	*Staphylococcus aureus* [122]	Protein A
RAB10, RAB20, RAB28 and RAB35	DNA	*Staphylococcus aureus* [123]	Whole bacteria
H1, H16, H4, L1, L10 and H19	DNA	*Streptococcus mutans* [124]	Whole bacteria
20A9, 20A24P, 20A9P, 20A12P, 20A14P and 15A3P	DNA	*Streptococcus pyogenes* [125]	M-Type bacteria
E-Cells 1, E-Cells 1P, E-CA 20, E-CA20P, D-Cells 9 and D-Cells9P	DNA	*Streptococcus pyogenes* [126]	Whole bacteria
VA2 and VA8	DNA	*Vibrio aliginolyticus* [127]	Whole bacteria
Ap1, Ap2, Apt3 and Apt4	DNA	*Vibrio parahaemolyticus* (ATCC 17802) [128]	Whole bacteria
Vapt2	DNA	*Vibrio vulnificus* [129]	Whole bacteria

Table 3 shows some of the current characterized aptamers to specific strains of bacteria and their specific targets.

## Abbreviations

AuNP	Gold nanoparticle
BSI	Bloodstream infection
CAUTI	Catheter-associated urinary tract infection
DNA	Deoxyribonucleic acid
ELISA	Enzyme-linked immune-sorbent assay
GI	Gastrointestinal
HAI	Hospital acquired infection
HCW	Health care worker
HIV	Human immunodeficiency virus
ICU	Intensive care unit
LFA	Lateral flow assay
MB	Methylene blue
MDR	Multi-drug resistant
MDRB	Multi-drug resistant bacteria
MRSA	Methicillin-resistance *S. aureus*
NGS	Next generation sequencing
PA	Protein A
PBP2a	Penicillin binding protein 2a
PCR	Polymerase chain reaction
POC	Point-of-care
RCA	Rolling circle amplification
SELEX	Systematic evolution of ligands via exponential enrichment
SSI	Surgical site infection
UTI	Urinary tract infection
VAP	Ventilator-associated pneumoniae
VRE	Vancomycin resistant enterococci

## References

[1] Cassir N., Thomas G., Hraiech S., Brunet J., Fournier P.-E., la Scola B., Papazian L. (2015). Chlorhexidine daily bathing: Impact on health care–associated infections caused by gram-negative bacteria. Am. J. Infect. Control..

[2] Vincent J.L., Rello J., Marshall J.K., Silva E., Anzueto A., Martin-Loeches I., Moreno R., Lipman J., Gomersall C., Sakr Y. (2009). International Study of the Prevalence and Outcomes of Infection in Intensive Care Units. JAMA.

[3] Graves N., Weinhold D., Tong E.N., Birrell F., Doidge S., Ramritu P., Halton K., Lairson D., Whitby M. (2007). Effect of Healthcare-Acquired Infection on Length of Hospital Stay and Cost. Infect. Control. Hosp. Epidemiol..

[4] Zhang C., Lv X., Han X., Man Y., Saeed Y., Qing H., Deng Y. (2015). Whole-cell based aptamer selection for selective capture of microorganisms using microfluidic devices. Anal. Methods.

[5] Jenkins D.R. (2017). Nosocomial infections, and infection control. Medicine.

[6] Tan R., Wang H., Li M., Huang J., Sun J., Qu H. (2014). Epidemiology and antimicrobial resistance among commonly encountered bacteria associated with infections and colonization in intensive care units in a university-affiliated hospital in Shanghai. J. Microbiol. Immunol. Infect..

[7] Khan H.A., Baig F.K., Mehboob R. (2017). Nosocomial infections: Epidemiology, prevention, control and surveillance. Asian Pac. J. Trop. Biomed..

[8] Khan H.A., Ahmad A., Mehboob R. (2015). Nosocomial infections and their control strategies. Asian Pac. J. Trop. Biomed..

[9] Cornejo-Juárez P., Vilar-Compte D., Pérez-Jiménez C., Ñamendys-Silva S., Sandoval-Hernández S., Volkow-Fernández P. (2015). The impact of hospital-acquired infections with multidrug-resistant bacteria in an oncology intensive care unit. Int. J. Infect. Dis..

[10] Le N.K., Hf W., Vu P.D., Khu D.T.K., Le H.T., Hoang B.T.N., Vo V.T., Lam Y.M., Vu D.T.V., Nguyen T.H. (2016). High prevalence of hospital-acquired infections caused by gram-negative carbapenem resistant strains in Vietnamese pediatric ICUs: A multi-centre point prevalence survey. Medicine.

[11] Arefian H., Hagel S., Heublein S., Rissner F., Scherag A., Brunkhorst F.M., Baldessarini R.J., Hartmann M. (2016). Extra length of stay and costs because of health care–associated infections at a German university hospital. Am. J. Infect. Control..

[12] Schmier J.K., Hulme-Lowe C.K., Semenova S., Klenk J.A., DeLeo P.C., Sedlak R., Carlson P.A. (2016). Estimated hospital costs associated with preventable health care-associated infections if health care antiseptic products were unavailable. Clin. Outcomes Res..

[13] Kaye K.S., Pogue J.M. (2015). Infections Caused by Resistant Gram-Negative Bacteria: Epidemiology and Management. Pharmacother. J. Hum. Pharmacol. Drug Ther..

[14] Royer S., Faria A.L.S., Seki L.M., Chagas T.P.G., de Campos P.A., Batistão D.W.D.F., Asensi M.D., Filho P.P.G., Ribas R.M. (2015). Spread of multidrug-resistant Acinetobacter baumannii and Pseudomonas aeruginosa clones in patients with ventilator-associated pneumonia in an adult intensive care unit at a university hospital. Braz. J. Infect. Dis..

[15] MacGowan A., Macnaughton E. (2017). Antibiotic resistance. Medicine.

[16] Kong L.Y., Dendukuri N., Schiller I., Bourgault A.-M., Brassard P., Poirier L., Lamothe F., Béliveau C., Michaud S., Turgeon N. (2015). Predictors of asymptomatic Clostridium difficile colonization on hospital admission. Am. J. Infect. Control.

[17] Longtin Y., Paquet-Bolduc B., Gilca R., Garenc C., Fortin E., Longtin J., Trottier S., Gervais P., Roussy J.-F., Lévesque S. (2016). Effect of Detecting and Isolating Clostridium difficile Carriers at Hospital Admission on the Incidence of C difficile Infections: A Quasi-Experimental Controlled Study. JAMA Intern. Med..

[18] Abt M.C., McKenney P.T., Pamer E.G. (2016). Clostridium difficile colitis: Pathogenesis and host defence. Nat. Rev. Genet..

[19] Dapa T., Unnikrishnan M. (2013). Biofilm formation byClostridium difficile. Gut Microbes.

[20] Thaden J.T., Li Y., Ruffin F., Maskarinec S.A., Hill-Rorie J.M., Wanda L.C., Reed S.D., Fowler V.G. (2016). Increased Costs Associated with Bloodstream Infections Caused by Multidrug-Resistant Gram-Negative Bacteria Are Due Primarily to Patients with Hospital-Acquired Infections. Antimicrob. Agents Chemother..

[21] Fisher R.A., Gollan B., Helaine S. (2017). Persistent bacterial infections and persister cells. Nat. Rev. Genet..

[22] Price L.B., Hungate B.A., Koch B.J., Davis G.S., Liu C.M. (2017). Colonizing opportunistic pathogens (COPs): The beasts in all of us. PLoS Pathog..

[23] Lockwood A.M., Perez K.K., Musick W.L., Ikwuagwu J.O., Attia E., Fasoranti O.O., Cernoch P.L., Olsen R.J., Musser J.M. (2016). Integrating Rapid Diagnostics and Antimicrobial Stewardship in Two Community Hospitals Improved Process Measures and Antibiotic Adjustment Time. Infect. Control. Hosp. Epidemiol..

[24] Templier V., Roux A., Roupioz Y., Livache T. (2016). Ligands for label-free detection of whole bacteria on biosensors: A review. TrAC Trends Anal. Chem..

[25] Vuotto C., Longo F., Balice M.P., Donelli G., Varaldo P.E. (2014). Antibiotic Resistance Related to Biofilm Formation in Klebsiella pneumoniae. Pathogens.

[26] Zhou Z., Hu B., Gao X., Bao R., Chen M., Li H. (2016). Sources of sporadic Pseudomonas aeruginosa colonizations/infections in surgical ICUs: Association with contaminated sink trap. J. Infect. Chemother..

[27] Brown S.P., Cornforth D.M., Mideo N. (2012). Evolution of virulence in opportunistic pathogens: Generalism, plasticity, and control. Trends Microbiol..

[28] Lefebvre A., Bertrand X., Quantin C., Vanhems P., Lucet J.C., Nuemi G., Astruc K., Chavanet P., Aho-Glélé L.S. (2017). Association between *Pseudomonas aeruginosa* positive water samples and healthcare-associated cases: Nine-year study at one university hospital. J. Hosp. Infect..

[29] Saxena S., Banerjee G., Garg R., Singh M. (2014). Comparative Study of Biofilm Formation in Pseudomonas aeruginosa Isolates from Patients of Lower Respiratory Tract Infection. J. Clin. Diagn. Res..

[30] Wang K.-Y., Zeng Y.-L., Yang X.-Y., Li W.-B., Lan X.-P. (2010). Utility of aptamer-fluorescence in situ hybridization for rapid detection of Pseudomonas aeruginosa. Eur. J. Clin. Microbiol. Infect. Dis..

[31] Cosgrove S.E., Qi Y., Kaye K.S., Harbarth S., Karchmer A.W., Carmeli Y. (2005). The Impact of Methicillin Resistance in Staphylococcus aureus Bacteremia on Patient Outcomes: Mortality, Length of Stay, and Hospital Charges. Infect. Control. Hosp. Epidemiol..

[32] Lin D., Ou Q., Lin J., Peng Y., Yao Z. (2017). A meta-analysis of the rates of Staphylococcus aureus and methicillin-resistant S aureus contamination on the surfaces of environmental objects that health care workers frequently touch. Am. J. Infect. Control..

[33] Falugi F., Kim H.K., Missiakas D.M., Schneewind O. (2013). Role of Protein A in the Evasion of Host Adaptive Immune Responses by Staphylococcus aureus. mBio.

[34] Inagaki K., Lucar J., Blackshear C., Hobbs C.V. (2019). Methicillin-susceptible and Methicillin-resistant Staphylococcus aureus Bacteremia: Nationwide Estimates of 30-Day Readmission, In-hospital Mortality, Length of Stay, and Cost in the United States. Clin. Infect. Dis..

[35] Baylay A.J., Piddock L.J., Webber M.A. (2019). Molecular Mechanisms of Antibiotic Resistance—Part I. Bact. Resist. Antibiot. Mol. Man.

[36] Murni I.K., Duke T., Kinney S., Daley A.J., Soenarto Y. (2015). Reducing hospital-acquired infections and improving the rational use of antibiotics in a developing country: An effectiveness study. Arch. Dis. Child..

[37] Zarpellon M.N., Gales A.C., Sasaki A.L., Selhorst G.J., Menegucci T.C., Cardoso C.L., Garcia L.B., Tognim M.C.B. (2015). Survival of vancomycin-intermediate *Staphylococcus aureus* on hospital surfaces. J. Hosp. Infect..

[38] Ling M.L., How K.B. (2013). Pseudomonas aeruginosa outbreak linked to sink drainage design. Heal. Infect..

[39] Durmaz G., Us T., Aydinli A., Kiremitci A., Kiraz N., Akgün Y. (2003). Optimum Detection Times for Bacteria and Yeast Species with the BACTEC 9120 Aerobic Blood Culture System: Evaluation for a 5-Year Period in a Turkish University Hospital. J. Clin. Microbiol..

[40] Kim H., Chung D.-R., Kang M. (2019). A new point-of-care test for the diagnosis of infectious diseases based on multiplex lateral flow immunoassays. Analyst.

[41] Khanal R., Sah P., Lamichhane P., Lamsal A., Upadhaya S., Pahwa V.K. (2015). Nasal carriage of methicillin resistant Staphylococcus aureus among health care workers at a tertiary care hospital in Western Nepal. Antimicrob. Resist. Infect. Control..

[42] Starlander G., Melhus Å. (2012). Minor outbreak of extended-spectrum β-lactamase-producing Klebsiella pneumoniae in an intensive care unit due to a contaminated sink. J. Hosp. Infect..

[43] Conly J., Johnston B. (2005). Where are all the new antibiotics? The new antibiotic paradox. Can. J. Infect. Dis. Med. Microbiol..

[44] Wu H., Moser C., Wang H.-Z., Høiby N., Song Z.-J. (2015). Strategies for combating bacterial biofilm infections. Int. J. Oral Sci..

[45] Seifi K., Kazemian H., Heidari H., Rezagholizadeh F., Saee Y., Shirvani F., Houri H. (2016). Evaluation of Biofilm Formation Among Klebsiella pneumoniae Isolates and Molecular Characterization by ERIC-PCR. Jundishapur J. Microbiol..

[46] Anderl J.N., Franklin M.J., Stewart P.S. (2000). Role of Antibiotic Penetration Limitation in Klebsiella pneumoniae Biofilm Resistance to Ampicillin and Ciprofloxacin. Antimicrob. Agents Chemother..

[47] Costerton J.W., Stewart P.S., Greenberg E.P. (1999). Bacterial Biofilms: A Common Cause of Persistent Infections. Science.

[48] Harms A., Maisonneuve E., Gerdes K. (2016). Mechanisms of bacterial persistence during stress and antibiotic exposure. Science.

[49] Harbarth S.J., Sax H., Gastmeier P. (2003). The preventable proportion of nosocomial infections: An overview of published reports. J. Hosp. Infect..

[50] Torres-Chavolla E., Alocilja E.C. (2009). Aptasensors for detection of microbial and viral pathogens. Biosens. Bioelectron..

[51] Byrne B., Stack E., Gilmartin N., O’Kennedy R.J. (2009). Antibody-Based Sensors: Principles, Problems and Potential for Detection of Pathogens and Associated Toxins. Sensors.

[52] Zowawi H.M., Harris P.N.A., Roberts M.J., Tambyah P.A., Schembri M.A., Pezzani M.D., Williamson D.A., Paterson D.L. (2015). The emerging threat of multidrug-resistant Gram-negative bacteria in urology. Nat. Rev. Urol..

[53] Foster T.J., Geoghegan J.A., Ganesh V.K., Höök M. (2014). Adhesion, invasion and evasion: The many functions of the surface proteins of Staphylococcus aureus. Nat. Rev. Genet..

[54] Yuling Z., Zhao Y., Liu C., Chen Z., Zhou D. (2014). Molecular pathogenesis ofKlebsiella pneumoniae. Futur. Microbiol..

[55] Stentzel S., Sundaramoorthy N., Michalik S., Nordengrün M., Schulz S., Kolata J., Kloppot P., Engelmann S., Steil L., Hecker M. (2015). Specific serum IgG at diagnosis of Staphylococcus aureus bloodstream invasion is correlated with disease progression. J. Proteom..

[56] Le T.T., Chang P., Benton D.J., McCauley J.W., Iqbal M., Cass A. (2017). Dual Recognition Element Lateral Flow Assay Toward Multiplex Strain Specific Influenza Virus Detection. Anal. Chem..

[57] Jayol A., Nordmann P., Desroches M., Decousser J.-W., Poirel L. (2016). Acquisition of Broad-Spectrum Cephalosporin Resistance Leading to Colistin Resistance in Klebsiella pneumoniae. Antimicrob. Agents Chemother..

[58] Verdoodt N., Basso C.R., Rossi B.F., Pedrosa V.A. (2017). Development of a rapid and sensitive immunosensor for the detection of bacteria. Food Chem..

[59] Gosiewski T., Ludwig-Galezowska A.H., Huminska K., Sroka-Oleksiak A., Radkowski P., Salamon D., Wojciechowicz J., Kus-Slowinska M., Bulanda M., Wołkow P.P. (2017). Comprehensive detection and identification of bacterial DNA in the blood of patients with sepsis and healthy volunteers using next-generation sequencing method - the observation of DNAemia. Eur. J. Clin. Microbiol. Infect. Dis..

[60] Ivnitski D., Abdel-Hamid I., Atanasov P., Wilkins E. (1999). Biosensors for detection of pathogenic bacteria. Biosens. Bioelectron..

[61] Sanvicens N., Pastells C., Pascual N., Marco M.-P. (2009). Nanoparticle-based biosensors for detection of pathogenic bacteria. TrAC Trends Anal. Chem..

[62] Ahmed A., Rushworth J.V., Hirst N.A., Millner P.A. (2014). Biosensors for Whole-Cell Bacterial Detection. Clin. Microbiol. Rev..

[63] Zhao Y., Wang H., Zhang P., Sun C., Wang X., Wang X., Yang R., Wang C., Zhou L. (2016). Rapid multiplex detection of 10 foodborne pathogens with an up-converting phosphor technology-based 10-channel lateral flow assay. Sci. Rep..

[64] Brosel-Oliu S., Ferreira R., Uria N., Abramova N., Gargallo R., Muñoz-Pascual F.-X., Bratov A. (2018). Novel impedimetric aptasensor for label-free detection of *Escherichia coli* O157:H7. Sens. Actuators B Chem..

[65] Paniel N., Baudart J., Hayat A., Barthelmebs L. (2013). Aptasensor and genosensor methods for detection of microbes in real world samples. Methods.

[66] Majdinasab M., Hayat A., Marty J. (2018). Aptamer-based assays and aptasensors for detection of pathogenic bacteria in food samples. TrAC Trends Anal. Chem..

[67] Hao L., Gu H., Duan N., Wu S., Ma X., Xia Y., Tao Z., Wang Z. (2017). An enhanced chemiluminescence resonance energy transfer aptasensor based on rolling circle amplification and WS2 nanosheet for Staphylococcus aureus detection. Anal. Chim. Acta.

[68] Bartholomew J.W., Mittwer T. (1952). The Gram stain. Bacteriol. Rev..

[69] Cother E.J., Vruggink H. (1980). Detection of viable and non-viable cells ofErwinia carotovora var.atroseptica in inoculated tubers of var. Bintje with enzyme-linked immunosorbent assay (ELISA). Potato Res..

[70] Steffan R.J., Atlas R.M. (1988). DNA amplification to enhance detection of genetically engineered bacteria in environmental samples. Appl. Environ. Microb..

[71] Fong W.K., Modrusan Z., McNevin J.P., Marostenmaki J., Zin B., Bekkaoui F. (2000). Rapid Solid-Phase Immunoassay for Detection of Methicillin-Resistant *Staphylococcus aureus* Using Cycling Probe Technology. J. Clin. Microbiol..

[72] Fratamico P., Strobaugh T., Medina M., Gehring A. (1998). Detection of *Escherichia coli* 0157:H7 using a surface plasmon resonance biosensor. Biotechnol. Tech..

[73] Bruno J.G., Kiel J.L. (1999). In vitro selection of DNA aptamers to anthrax spores with electrochemiluminescence detection. Biosens. Bioelectron..

[74] Wurtzel O., Dori-Bachash M., Pietrokovski S., Jurkevitch E., Sorek R. (2010). Mutation Detection with Next-Generation Resequencing through a Mediator Genome. PLoS ONE.

[75] Ahmed N., Dobrindt U., Hacker J., Hasnain S.E. (2008). Genomic fluidity and pathogenic bacteria: Applications in diagnostics, epidemiology and intervention. Nat. Rev. Genet..

[76] Felföldi T., Heéger Z., Vargha M., Márialigeti K. (2010). Detection of potentially pathogenic bacteria in the drinking water distribution system of a hospital in Hungary. Clin. Microbiol. Infect..

[77] Zou Y., Liang J., She Z., Kraatz H. (2019). Gold nanoparticles-based multifunctional nanoconjugates for highly sensitive and enzyme-free detection of E. coli K12. Talanta.

[78] Zelada-Guillén G.A., Sebastián-Avila J.L., Blondeau P., Riu J., Rius F.X. (2012). Label-free detection of Staphylococcus aureus in skin using real-time potentiometric biosensors based on carbon nanotubes and aptamers. Biosens. Bioelectron..

[79] Ferguson C., Booth N., Allan E. (2000). An ELISA for the detection of Bacillus subtilis L-form bacteria confirms their symbiosis in strawberry. Lett. Appl. Microbiol..

[80] Králík P., Ricchi M. (2017). A Basic Guide to Real Time PCR in Microbial Diagnostics: Definitions, Parameters, and Everything. Front. Microbiol..

[81] Kinghorn A.B., Dirkzwager R.M., Liang S., Cheung Y.-W., Fraser L.A., Shiu S.C.-C., Tang M.S.L., Tanner J.A. (2016). Aptamer Affinity Maturation by Resampling and Microarray Selection. Anal. Chem..

[82] Brody E.N., Gold L. (2000). Aptamers as therapeutic and diagnostic agents. Rev. Mol. Biotechnol..

[83] Toh S.Y., Citartan M., Gopinath S.C., Tang T.-H. (2015). Aptamers as a replacement for antibodies in enzyme-linked immunosorbent assay. Biosens. Bioelectron..

[84] Tombelli S., Minunni M., Mascini M. (2007). Analytical applications of aptamers. Internat. Congr. Opt. Optoelectron..

[85] Cerchia L., de Franciscis V. (2010). Targeting cancer cells with nucleic acid aptamers. Trends Biotechnol..

[86] Shigdar S., Qian C., Lv L., Pu C., Li Y., Li L., Marappan M., Lin J., Wang L., Duan W. (2013). The Use of Sensitive Chemical Antibodies for Diagnosis: Detection of Low Levels of Epcam in Breast Cancer. PLoS ONE.

[87] Song M.Y., Nguyen D., Hong S.W., Kim B.C. (2017). Broadly reactive aptamers targeting bacteria belonging to different genera using a sequential toggle cell-SELEX. Sci. Rep..

[88] Hanif A., Farooq R., Rehman M.U., Khan R., Majid S., Ganaie M.A. (2018). Aptamer based nanobiosensors: Promising healthcare devices. Saudi Pharm. J..

[89] Griffiths A.D., Duncan A.R. (1998). Strategies for selection of antibodies by phage display. Curr. Opin. Biotechnol..

[90] Bu T., Yao X., Huang L., Dou L., Zhao B., Yang B., Li T., Wang J., Zhang D. (2020). Dual recognition strategy and magnetic enrichment based lateral flow assay toward Salmonella enteritidis detection. Talanta.

[91] Xu L., Dai Q., Shi Z., Liu X., Gao L., Wang Z., Zhu X., Li Z. (2020). Accurate MRSA identification through dual-functional aptamer and CRISPR-Cas12a assisted rolling circle amplification. J. Microbiol. Methods.

[92] Gürtler V. (2015). Predicting genome variations between passages of Clostridium difficle by ribotypes. Microbiol. Aust..

[93] Bourgeois I., Camiade E., Biswas R., Courtin P., Gibert L., Götz F., Chapot-Chartier M.-P., Pons J.-L., Pestel-Caron M. (2009). Characterization of AtlL, a bifunctional autolysin of Staphylococcus lugdunensis with N-acetylglucosaminidase and N-acetylmuramoyl-l-alanine amidase activities. FEMS Microbiol. Lett..

[94] Tominaga T. (2018). Rapid detection of Klebsiella pneumoniae, Klebsiella oxytoca, Raoultella ornithinolytica and other related bacteria in food by lateral-flow test strip immunoassays. J. Microbiol. Methods.

[95] Kim J., Campbell A.S., de Ávila B.E., Wang J. (2019). Wearable biosensors for healthcare monitoring. Nat. Biotechnol..

[96] Scharinger E.J., Dietrich R., Wittwer T., Märtlbauer E., Schauer K. (2017). Multiplexed Lateral Flow Test for Detection and Differentiation of Cronobacter sakazakii Serotypes O1 and O2. Front. Microbiol..

[97] Wang R., Kim K., Choi N., Wang X., Lee J., Jeon J.H., Rhie G.-E., Choo J. (2018). Highly sensitive detection of high-risk bacterial pathogens using SERS-based lateral flow assay strips. Sens. Actuators B Chem..

[98] Su L., Jia W., Hou C., Lei Y. (2011). Microbial biosensors: A review. Biosens. Bioelectron..

[99] Zhang D., Liu Q. (2016). Biosensors and bioelectronics on smartphone for portable biochemical detection. Biosens. Bioelectron..

[100] Bang G.S., Cho S., Kim B.-G. (2005). A novel electrochemical detection method for aptamer biosensors. Biosens. Bioelectron..

[101] Mehrotra P. (2016). Biosensors and their applications—A review. J. Oral Biol. Craniofacial Res..

[102] Song S., Wang L., Li J., Fan C., Zhao J. (2008). Aptamer-based biosensors. TrAC Trends Anal. Chem..

[103] Hamula C.L.A., Zhang H., Guan L.L., Li X.-F., Le X.C. (2008). Selection of Aptamers against Live Bacterial Cells. Anal. Chem..

[104] Gopinath S.C.B., Lakshmipriya T., Chen Y., Phang W.-M., Hashim U. (2016). Aptamer-based ‘point-of-care testing’. Biotechnol. Adv..

[105] Zou Y., Duan N., Wu S., Shen M., Wang Z. (2018). Selection, Identification, and Binding Mechanism Studies of an ssDNA Aptamer Targeted to Different Stages of *E. coli* O157:H7. J. Agric. Food Chem..

[106] White R., Rusconi C.P., Scardino E., Wolberg A.S., Lawson J.H., Hoffman M., A Sullenger B. (2001). Generation of Species Cross-reactive Aptamers Using “Toggle” SELEX. Mol. Ther..

[107] la Housse M., Park H.-C., Lee S.-C., Ha N.-R., Jung I.-P., Schlesinger S.R., Shackelford K., Yoon M.-Y., Kim S.J. (2018). Inhibition of anthrax lethal factor by ssDNA aptamers. Arch. Biochem. Biophys..

[108] Biondi E., Lane J.D., Das D., Dasgupta S., Piccirilli J.A., Hoshika S., Bradley K.M., Krantz B.A., Benner S.A. (2016). Laboratory evolution of artificially expanded DNA gives redesignable aptamers that target the toxic form of anthrax protective antigen. Nucleic Acids Res..

[109] Dwivedi H.P., Smiley R.D., Jaykus L.-A. (2010). Selection and characterization of DNA aptamers with binding selectivity to Campylobacter jejuni using whole-cell SELEX. Appl. Microbiol. Biotechnol..

[110] Marton S., Cleto F., Krieger M.A., Cardoso J. (2016). Isolation of an Aptamer that Binds Specifically to E. coli. PLoS ONE.

[111] Renders M., Miller E., Lam C.H., Perrin D. (2017). Whole cell-SELEX of aptamers with a tyrosine-like side chain against live bacteria. Org. Biomol. Chem..

[112] Amraee M., Oloomi M., Yavari A., Bouzari S. (2017). DNA aptamer identification and characterization for E. coli O157 detection using cell-based SELEX method. Anal. Biochem..

[113] Yan W., Gu L., Ren W., Ma X., Qin M., Lyu M., Wang S. (2019). Recognition of Helicobacter pylori by protein-targeting aptamers. Helicobacter.

[114] Graziani A.C., Stets M.I., Lopes A.L.K., Schluga P.H.C., Marton S., Mendes I.F., de Andrade A.S.R., Krieger M.A., Cardoso J. (2017). High Efficiency Binding Aptamers for a Wide Range of Bacterial Sepsis Agents. J. Microbiol. Biotechnol..

[115] Mozioglu E., Gokmen O., Tamerler C., Kocagoz Z.T., Akgoz M. (2015). Selection of Nucleic Acid Aptamers Specific for Mycobacterium tuberculosis. Appl. Biochem. Biotechnol..

[116] Zimbres F.M., Tárnok A., Ulrich H.D., Wrenger C. (2013). Aptamers: Novel Molecules as Diagnostic Markers in Bacterial and Viral Infections?. BioMed Res. Int..

[117] Soundy J., Day D.J. (2017). Selection of DNA aptamers specific for live Pseudomonas aeruginosa. PLoS ONE.

[118] Duan N., Wu S., Chen X., Huang Y., Xia Y., Ma X., Wang Z. (2013). Selection and Characterization of Aptamers against Salmonella typhimurium Using Whole-Bacterium Systemic Evolution of Ligands by Exponential Enrichment (SELEX). J. Agric. Food Chem..

[119] Sedighian H., Halabian R., Amani J., Heiat M., Amin M., Fooladi A.A.I. (2018). Staggered Target SELEX, a novel approach to isolate non-cross-reactive aptamer for detection of SEA by apta-qPCR. J. Biotechnol..

[120] Wang K., Gan L., Jiang L., Zhang X., Yang X., Chen M., Lan X. (2015). Neutralization of Staphylococcal Enterotoxin B by an Aptamer Antagonist. Antimicrob. Agents Chemother..

[121] Stoltenburg R., Krafčiková P., Víglaský V., Strehlitz B. (2016). G-quadruplex aptamer targeting Protein A and its capability to detect Staphylococcus aureus demonstrated by ELONA. Sci. Rep..

[122] Stoltenburg R., Strehlitz B. (2018). Refining the Results of a Classical SELEX Experiment by Expanding the Sequence Data Set of an Aptamer Pool Selected for Protein A. Int. J. Mol. Sci..

[123] Ramlal S., Mondal B., Lavu P.S., Kingston J. (2018). Capture and detection of Staphylococcus aureus with dual labeled aptamers to cell surface components. Int. J. Food Microbiol..

[124] Cui W., Liu J., Su D., Hu D., Hou S., Hu T., Yang J., Luo Y., Xi Q., Chu B. (2016). Identification of ssDNA aptamers specific to clinical isolates of Streptococcus mutans strains with different cariogenicity. Acta Biochim. Biophys. Sin..

[125] Hamula C.L., Le X.C., Li X.-F. (2011). DNA Aptamers Binding to Multiple Prevalent M-Types ofStreptococcus pyogenes. Anal. Chem..

[126] Hamula C.L., Peng H., Wang Z., Tyrrell G.J., Li X.-F., Le X.C. (2016). An improved SELEX technique for selection of DNA aptamers binding to M-type 11 of Streptococcus pyogenes. Methods.

[127] Yu Q., Liu M., Su H., Xiao H., Wu S., Qin X., Li S., Mi H., Lu Z., Shi D. (2019). Selection and characterization of ssDNA aptamers specifically recognizing pathogenic Vibrio alginolyticus. J. Fish. Dis..

[128] Song S., Wang X., Xu K., Li Q., Ning L., Yang X. (2019). Selection of highly specific aptamers to Vibrio parahaemolyticus using cell-SELEX powered by functionalized graphene oxide and rolling circle amplification. Anal. Chim. Acta.

[129] Yan W., Gu L., Liu S., Ren W., Lyu M., Wang S. (2018). Identification of a highly specific DNA aptamer for Vibrio vulnificus using systematic evolution of ligands by exponential enrichment coupled with asymmetric PCR. J. Fish. Dis..

[130] Yan A.C., Levy M. (2009). Aptamers and aptamer targeted delivery. RNA Biol..

[131] Becker S., Theile S., Heppeler N., Michalczyk A., Wentzel A., Wilhelm S., Jaeger K.-E., Kolmar H. (2005). A generic system for the Escherichia coli cell-surface display of lipolytic enzymes. FEBS Lett..

[132] Anis E., Hawkins I.K., Ilha M.R.S., Woldemeskel M.W., Saliki J.T., Wilkes R.P. (2018). Evaluation of Targeted Next-Generation Sequencing for Detection of Bovine Pathogens in Clinical Samples. J. Clin. Microbiol..

[133] Motro Y., Moran-Gilad J. (2017). Next-generation sequencing applications in clinical bacteriology. Biomol. Detect. Quantif..

[134] Udugama B., Kadhiresan P., Kozlowski H.N., Malekjahani A., Osborne M., Li V.Y.C., Chen H., Mubareka S., Gubbay J.B., Chan W.C.W. (2020). Diagnosing COVID-19: The Disease and Tools for Detection. ACS Nano.

[135] Villalonga A., Pérez-Calabuig A.M., Villalonga R. (2020). Electrochemical biosensors based on nucleic acid aptamers. Anal. Bioanal. Chem..

